# Computational method for the identification of third generation activity cliffs

**DOI:** 10.1016/j.mex.2020.100793

**Published:** 2020-01-14

**Authors:** Dagmar Stumpfe, Huabin Hu, Jürgen Bajorath

**Affiliations:** Department of Life Science Informatics, B-IT, LIMES Program Unit Chemical Biology and Medicinal Chemistry, Rheinische Friedrich-Wilhelms-Universität, Endenicher Allee 19c, D-53115 Bonn, Germany

**Keywords:** Third generation activity cliff identification, Compound structure and activity data, Computational analysis, Molecular similarity, Analog pairs, Potency value distributions, Potency differences, Substitution sites, Single-site activity cliffs, Multi-site activity cliffs, Structure-activity relationships

## Abstract

In medicinal chemistry and chemoinformatics, activity cliffs (ACs) are defined as pairs of structurally similar compounds that are active against the same target but have a large difference in potency. Accordingly, ACs are rich in structure-activity relationship (SAR) information, which rationalizes their relevance for medicinal chemistry. For identifying ACs, a compound similarity criterion and a potency difference criterion must be specified. So far a constant potency difference between AC partner compounds has mostly been set, e.g. 100-fold, irrespective of the specific activity (targets) of cliff-forming compounds. Herein, we introduce a computational methodology for AC identification and analysis that includes three novel components:

• ACs are identified on the basis of variable target set-dependent potency difference criteria (a ‘target set’ represents a collection of compounds that are active against a given target protein).
• ACs are extracted from computationally determined analog series (ASs) and consist of pairs of analogs with single or multiple substitution sites.
• For multi-site ACs, a search for analogs with individual substitutions is performed to analyze their contributions to AC formation and determine if multi-site ACs can be represented by single-site ACs.

## Graphical abstract

**Figure:**
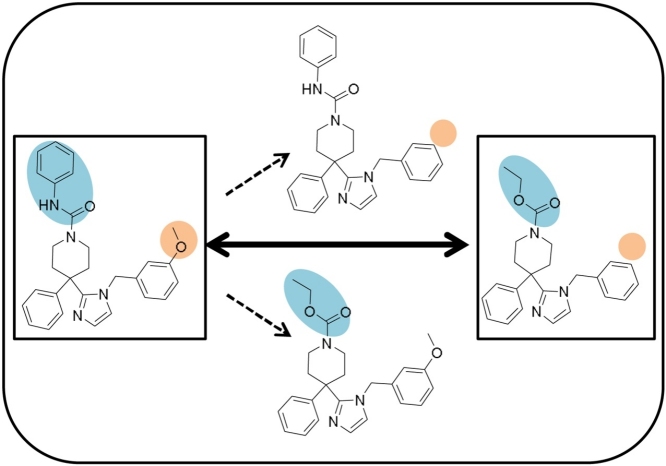

**Specification Table**

**Table:** 

Subject area:	Chemistry
More specific subject area:	Computational medicinal chemistry
Method name:	Third generation activity cliff identification
Name and reference of original method:	H. Hu, D. Stumpfe, J. Bajorath, Second-generation activity cliffs identified on the basis of target set-dependent potency difference criteria, Future Med. Chem. 11 (2019) 379-394. D. Stumpfe, H. Hu, J. Bajorath, Introducing a new category of activity cliffs with chemical modifications at multiple sites and rationalizing contributions of individual substitutions, Bioorg. Med. Chem. 27 (2019) 3605-3612.
Resource availability:	https://doi.org/10.5281/zenodo.1436584 H. Hu, D. Stumpfe, J. Bajorath, Systematic identification of target set-dependent activity cliffs. Future Sci. OA 5 (2019) FSO363.

## Method details

### AC categories

Considering different ways in which the compound similarity and potency difference criterion can be specified and applied for AC definition, the following AC categories are introduced:

*First generation* ACs: ACs defined on the basis of a constantly applied similarity criterion, i.e., Tanimoto similarity or substructure-based similarity [1], and a constant potency difference criterion [2], irrespective of the target sets under study.

*Second generation* ACs: ACs defined on the basis of a constant substructure-based similarity criterion and a variable target set-dependent potency difference criterion [3].

*Third generation* ACs: ACs formed by pairs of structural analogs with single or multiple substitution sites extracted from individual ASs and applying target set-dependent potency difference thresholds [4].

### Methodological framework

The method for the identification of *third generation ACs* combines methodological components from Hu et al. [3] and Stumpfe et al. [4] and makes use of the following computational concepts:

i ASs were identified using the compound-core relationship (CCR) methodology [5]. Following the CCR approach, exocyclic single bonds in test compounds are systematically fragmented according to retrosynthetic combinatorial analysis procedure (RECAP) rules [6] permitting a maximum of five fragmentation sites per compound. Each of these sites represents a possible substitution site. Accordingly, each fragmentation step yields a compound core and a substituent. A core is required to have at least twice the size of the substituent or combined multiple substituents (i.e., twice the number of non-hydrogen atoms). For each test compound, all possible core-fragment combinations with one to five substitution sites are sampled and substituents at each site are replaced by a hydrogen atom to generalize the core representation. Then, all compounds sharing the same core are combined representing an individual AS [5]. It follows that compounds belonging to the same AS are distinguished by modifications at a single or multiple substitution sites. In addition, search calculations for analogs having individual substitutions found in ACs with multiple substitution sites were carried out with the aid of the OpenEye chemistry toolkit [7]
ii As substructure-based similarity criteria for ACs, our group developed a preference for the formation of matched molecular pairs (MMPs) [8,9] with size-restricted substituents [10] (*second generation ACs*) and pairs of analogs from the same AS [4] (*third generation ACs*). An MMP is defined as a pair of compounds that only differ by a structural modification (substitution) at a single site [8]. To systematically generate MMPs, single bonds in compounds can be randomly fragmented [9] or on the basis of RECAP rules, yielding retrosynthetic MMPs (RMMPs) [11]. The resulting ACs are termed MMP-cliffs [10] and RMMP-cliffs [12], respectively. The application of MMPs and RMMPs as a similarity criterion yields ACs with only a single substitution site.
iii Compound potency distributions in target sets are analyzed in boxplots and the interquartile range (IQR) is determined [12]. The IQR represents the potency range between quartile 1 (Q1) and 3 (Q3) for ∼50 % of the compounds in a set. For AC analysis, only target sets with an IQR of at least one order of magnitude are selected because sets with a smaller IQR value rarely contain ACs [12].

### Activity data requirements

To ensure accuracy of AC assignments, only compounds with confirmed specific activity against a given target and numerically defined (assay-independent) equilibrium constants (pK_i_ values) are considered.

### Method design

**Fig. 1:**
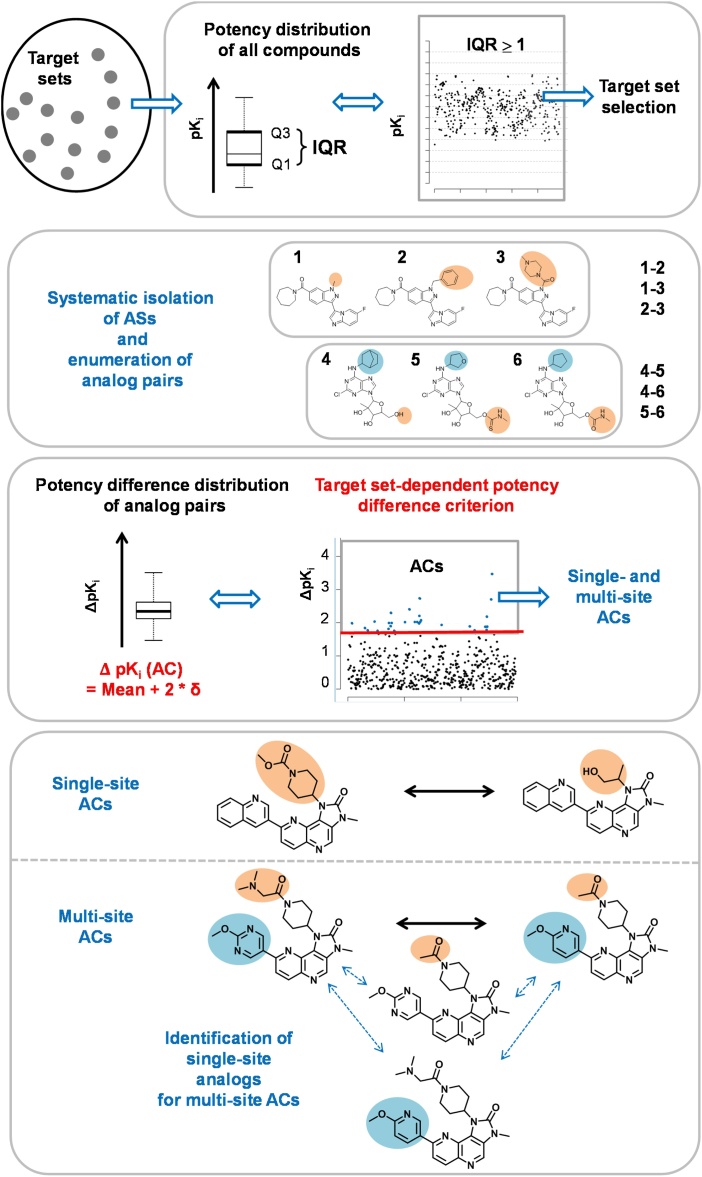
Summary of the computational methodology for the identification of *third generation ACs* in target sets. Boxes present different steps of the approach.

Fig. 1 provides a schematic summary of the different steps involved in computationally identifying and analyzing *third generation ACs* (from the top to the bottom), as detailed in the following:

*1.0.* Target sets are pre-selected on the basis of variable compound potency distributions (IQR ≥ 1) that typically yield ACs.

*2.1.* From a qualifying target set, ASs are systematically extracted. Compounds comprising each AS share a common core and contain single or multiple substitution sites.

*2.2*. For each AS, all analog pairs are enumerated and the potency difference captured by each pair is calculated. The formation of an analog pair with a single or multiple substitution sites serves as a *similarity criterion* for AC formation.

*3.1.* For each target set, all analog pairs (from all ASs) are collected and their potency difference distribution is determined.

*3.2.* As potency difference criterion for AC formation, the value of the mean of the distribution plus two standard deviations (sigma) is used.

*4.1*. All single- and multi-site ACs are collected (*third generation* ACs).

*4.2.* For multi-site ACs, a search for single-site analogs is carried out that contain individual substitutions and make it possible to study their contributions to AC formation, thus further characterizing multi-site ACs.

### Method validation

*Second* and *third generation ACs* were systematically extracted from target set available in ChEMBL [13], the major public repository of compounds and activity data from medicinal chemistry. From ChEMBL release 23, a total of 16,096 target set-dependent RMMP-cliffs were extracted that originated from 212 different target sets [3]. In addition, on the basis of ChEMBL release 24.1, a collection of 13,546 target set-dependent MMP-cliffs and 7995 set-dependent RMMP-cliffs was generated and made publicly available in an open access deposition to enable follow-up investigations of *second generation ACs* [14]. Furthermore, in ChEMBL release 24.1, a total of 16,454 *third generation ACs* were detected that originated from 209 target sets. These ACs included 12,249 instances with a single and 4205 instances with multiple substitution sites, 3805 of which were dual-site ACs [4]. Analog search identified both single-site analogs for 297 dual-site ACs that contained individual substitutions and revealed their potency effects. The potency difference of a subset of 141 dual-site ACs was determined by one of two substitutions. By contrast, in 156 cases, both substitutions were found to significantly contribute. These 156 confirmed dual-site ACs made it possible to study substitution-associated potency effects in greater detail, revealing additive, synergistic, and compensatory effects of individual substitutions [4].
